# Family history of any cancer for childhood leukemia patients in Sweden

**DOI:** 10.1002/jha2.166

**Published:** 2021-05-03

**Authors:** Xinjun Li, Kristina Sundquist, Jan Sundquist, Asta Försti, Kari Hemminki

**Affiliations:** ^1^ Center for Primary Health Care Research Lund University Malmö Sweden; ^2^ Department of Family Medicine and Community Health Department of Population Health Science and Policy Icahn School of Medicine at Mount Sinai New York New York; ^3^ Center for Community‐based Healthcare Research and Education (CoHRE) Department of Functional Pathology School of Medicine Shimane University Shimane Japan; ^4^ Hopp Children's Cancer Center (KiTZ) Heidelberg Germany; ^5^ Division of Pediatric Neurooncology German Cancer Research Center (DKFZ) German Cancer Consortium (DKTK) Heidelberg Germany; ^6^ Faculty of Medicine and Biomedical Center in Pilsen Charles University in Prague Pilsen Czech Republic; ^7^ Division of Cancer Epidemiology German Cancer Research Centre (DKFZ) Heidelberg Germany

**Keywords:** cancer registry, familial risk, family database, leukemia, susceptibility genes

## Abstract

Acute lymphoblastic leukemia (ALL) is the most common childhood leukemia, while the other types, acute myeloid leukemia (AML), chronic lymphocytic leukemia (CLL), and chronic myeloid leukemia (CML) are much rarer. While data on familial risks for childhood ALL have been emerging, such data for the other childhood leukemias are hardly available. We aim to fill in the gap of knowledge by assessing familial clustering of each childhood leukemia with childhood and adult leukemia and with any cancer. We identified 4461 childhood leukemias from the Swedish Cancer Registry and obtained their family members from the Multigeneration Register. Standardized incidence ratios (SIRs) were 3.34 for singleton siblings both diagnosed with ALL before age 20 years and 1.64 for those who had a family member diagnosed with ALL in adult age. Other childhood leukemias showed no familial risk, but childhood ALL risk was increased to 1.40 when adult family members were diagnosed with CLL. Childhood ALL was associated with endometrial cancer, and female ALL patients showed increased risk when family members were diagnosed with testicular cancer, melanoma, and skin squamous cell carcinoma. Childhood CLL was associated with rectal cancer, and childhood AML was associated with pancreatic and bladder cancers. As most of these associations are reported for the first time, there is a need to replicate the findings from independent sources.

## 1 INTRODUCTION

Acute lymphoblastic leukemia (ALL) is the most common childhood leukemia, followed by acute myeloid leukemia (AML) with an incidence of one fifth of that of ALL; chronic lymphocytic and myeloid leukemias (CLL, CML) are rare [1]. ALL is unique among childhood leukemias that the peak incidence occurs within the first years after birth which is suggested to indicate the in utero origin of this disease [1, 2]. In the clinical presentation of ALL, precursor B cells appear in blood and populate the bone marrow [3]. In older children, the proportion of T‐cell ALL increases and in adults (past age 20 years, about 40% of all ALL) it accounts for 25% of the total [4]. Recurrent cytogenetic abnormalities are found in 90% of children and 70% of adults, and fusion genes are common in patients of any age [5, 6, 7]. Cytotoxic therapies, which are effective in childhood ALL, are generally ineffective in the management of adult ALL, for which the long‐term survival remains relatively poor [8, 9]. This is in part explained by the reduced frequency of genetic alterations associated with favorable outcome in children, such as high hyperdiploidy and ETV6‐RUNX1, and a rising incidence of adverse genetic alterations, such as the Philadelphia chromosome (BCR‐ABL1 fusion), which is far more common in adults (15‐30%) than in children (2%) [4, 5, 6, 7].

Few environmental risk factors have been identified for childhood ALL including ionizing radiation, chemotherapy regimens, undefined viral infections, and high parental age [10, 11, 12, 13]. Familial risk between siblings is also a known rare risk factor; the risk is particular high among monozygotic twins where sharing of blood cells during pregnancy has been explained as the mechanism [10, 14, 15]. Although earlier considered largely a nonhereditary disease, a growing number of predisposing genetic alterations are revealed in familial and sporadic ALL [3]. The related gene variants are generally very rare and include a number of functional pathways including DNA repair, immunodeficiency, tumor suppression, and Down syndrome [3, 16]. Some of the predisposing genes or syndromes (RUNX1, ETV1, and Down syndrome) are also risk factors for other hematological neoplasms but many others predispose also to solid tumors (ATM, BLM, Fanconi anemia genes, TP53, NF1, and mismatch repair genes) [3]. Some common somatic gene defects are used as important clinical and prognostic disease classifiers including PAX5, ETV6, and IKZF1 [3]. The 2016 revision to the World Health Organization classification of myeloid neoplasms and acute leukemia considered a special section on “myeloid neoplasms with germ line predisposition” [17]. Large genome‐wide association studies (GWASs) have identified increasing numbers of common low‐risk gene variants in the germline; some of these map to the loci of the pathogenic high‐risk genes, and include the above three genes. The common low‐risk variants constitute a polygenic population risk continuum of individuals with numbers of risk alleles ranging from low to high [18, 19, 20]. Some of the identified low‐risk genes manifest pleiotropic effects in other hematological neoplasms [21]. In the most recent study, 16 significant loci were identified accounting for a joint heritability of 21% [19]. Nevertheless, old population‐level family studies have not been able to demonstrate associations of ALL with other hematological or solid tumors [14, 22]. Apart from low patients numbers and limited statistical power, the reasons may be a limited sharing of predisposing genes between ALL and other cancers. Familial risks of adult leukemias and other other hematological malignancies have been published earlier [23, 24].

In the present study, we used the unique Swedish population data resources to assess familial clustering of ALL and other childhood leukemias (diagnosed before age 20 years) concordantly (with the same leukemia in adults) and discordantly (with a different leukemia in adults). Additionally, we search for evidence on familial risk for childhood cancer with any other cancer. The family relationships were obtained from a complete population register at Statistics Sweden and all cancer data were derived from the Swedish Cancer Registry, guaranteeing reliability of the data.

## 2 MATERIALS AND METHODS

For the Swedish Family‐Cancer Database, family relationships were obtained from the Multigeneration Register, containing the Swedish population in families. “The offspring generation” was born after 1931 and by year 2015 oldest offspring reached age 83 years; siblings could be defined only in the offspring generation. Cancer data were identified from the Swedish Cancer Registry which was started in 1958 using codes of the International Classification of Diseases version 7 and later revisions. Information from the registers was linked at the individual level via the national 10‐digit civic registration number. In the linked dataset, civic registration numbers were replaced with serial numbers to ensure anonymity.

Familial risk was considered for offspring with childhood leukemia whose first‐degree relatives (parent or siblings) or more distant relatives were diagnosed with the same (concordant) or different (discordant) cancer; the relatives were thus probands. Standardized incidence ratios (SIRs) were calculated as the ratio of observed to expected number of cases. The expected numbers were calculated for all individuals without cancer in family members, and the rates were standardized by 5‐year age, gender, period (5 years group), socioeconomic status, and residential area. The expected rates were derived from the present dataset covering the Swedish population. As the SIR calculation was based on person‐years at risk, it was independent of family size. However, if the first child of the family is affected and if he will have fewer siblings, the likelihood of observing an affected sibling pair would reduce [25]. The 95% confidence interval (95%CI) of the SIR was calculated assuming a Poisson distribution. Observed cases (O) indicate the persons whom the SIR was calculated. Twins were analyzed separately and excluded from the overall analyses.

## 3 RESULTS

The characteristics of the study population are shown in Supporting information Table S1. The total index population at age below 20 years amounted to 8.5 million individuals recorded from year 1938 onwards. Leukemia was diagnosed in 4461 individuals, 55.3% boys and 44.3% diagnosed before age 5 years. Undefined leukemia was diagnosed in 916 patients; ALL was diagnosed in 2859 (80.6% of defined leukemias) children, followed by AML in 556 children (15.5%).

Among childhood leukemia patients, three pairs of twins were identified both of whom were diagnosed with ALL in childhood. They were all females with an SIR of 119.76 (95% CI, 57.03‐221.09). There were additionally three female twins diagnosed with ALL (SIR 2.46, 0.89‐5.40), whose cotwins were diagnosed each with CLL, AML, and unspecified leukemia. Because of the high‐risks twins were not included in the remaining analyses.

Familial risks for the four childhood leukemias are shown in Table 1 when first‐degree relatives were diagnosed with the same leukemia at age before 20 years or at a higher age. A total of 32 children were diagnosed with ALL (SIR 1.92) when family members were diagnosed with ALL at any age. The SIR for concordant childhood ALL was 3.34. Even in this largest group of leukemias, the case numbers were so few that no age‐ or sex‐group comparison was significant (95% CIs overlapped). For other leukemias, almost all probands were adult patients and no risk reached statistical significance. Among ALL patients, 41 patients (including twins) had a family history of ALL, that is, 1.4% (41/2859).

**TABLE 1:** Familial SIRs for concordant leukemia diagnosed in male and female patients before age 20 years

	Males				Females				All			
Subtypes of leukemia	O	SIR	95% CI		O	SIR	95% CI		O	SIR	95% CI	
Acute lymphoblastic leukemia	20	**2.16**	**1.32**	**3.34**	12	1.62	0.83	2.83	32	**1.92**	**1.31**	**2.71**
Family with leukemia < 20 years	6	**3.97**	**1.43**	**8.70**	3	2.54	0.48	7.51	9	**3.34**	**1.51**	**6.37**
Family with leukemia ≥ 20 years	14	1.80	0.98	3.04	9	1.44	0.65	2.75	23	**1.64**	**1.04**	**2.47**
Chronic lymphocytic leukemia	0				2	9.19	0.87	33.79	2	2.91	0.27	10.71
Family with leukemia < 20 years	0				0				0			
Family with leukemia ≥ 20 years	0				2	9.55	0.90	35.13	2	3.01	0.28	11.06
Acute myeloid leukemia	15	1.30	0.73	2.16	14	1.36	0.74	2.28	29	1.33	0.89	1.91
Family with leukemia < 20 years	1	2.61	0.00	14.97	0				1	1.38	0.00	7.92
Family with leukemia ≥ 20 years	14	1.26	0.69	2.12	14	1.40	0.77	2.36	28	1.33	0.88	1.92
Chronic myeloid leukemia	1	0.40	0.00	2.30	2	1.15	0.11	4.24	3	0.71	0.13	2.10
Family with leukemia < 20 years	0				0				0			
Family with leukemia ≥ 20 years	1	0.41	0.00	2.37	2	1.56	0.15	5.75	3	0.81	0.15	2.40

CI,  confidence interval; O, observed; SIR,  Standardized incidence ratio.

Bolding indicates that that 95%CI does not include SIR 1.00.

Familial risk for childhood ALL was assessed when family members were diagnosed with other leukemia (Table 2). Only CLL showed a significant association when adult parents were affected (SIR 1.40).

**TABLE 2:** SIRs for acute lymphoblastic leukemia (ALL) diagnosed before age 20 years when family members were diagnosed with discordant leukemia

	Males ALL				Females ALL				All ALL			
Subtypes of leukemia in family	O	SIR	95% CI		O	SIR	95% CI		O	SIR	95% CI	
Chronic lymphocytic leukemia	23	1.25	0.79	1.88	23	**1.59**	**1.01**	**2.39**	46	**1.40**	**1.03**	**1.87**
Family diagnosed < 20 years	0				0				0			
Family diagnosed ≥ 20 years	23	1.25	0.79	1.88	23	**1.59**	**1.01**	**2.39**	46	**1.40**	**1.03**	**1.87**
Acute myeloid leukemia	15	1.22	0.68	2.01	15	1.53	0.86	2.53	30	1.36	0.92	1.94
Family diagnosed < 20 years	0				1	4.86	0.00	27.84	1	2.15	0.00	12.34
Family diagnosed ≥ 20 years	15	1.24	0.69	2.06	14	1.46	0.80	2.46	29	1.34	0.90	1.93
Chronic myeloid leukemia	4	0.69	0.18	1.78	2	0.43	0.04	1.58	6	0.57	0.21	1.26
Family diagnosed < 20 years	0				0				0			
Family diagnosed ≥ 20 years	4	0.70	0.18	1.80	2	0.43	0.04	1.59	6	0.58	0.21	1.27

ALL, acute lymphoblastic leukemia; CI, confidence intervals; O, observed; SIR, standardized incidence ratio.

Bolding indicates that that 95%CI does not include SIR 1.00.

Associations according to the type of family relationship were tested (Table 3). For ALL, significant associations for childhood ALL were noted for siblings (3.32) and cousins (1.88). For CLL, the association was with grandparents (1.48) for which relationship the case numbers were largest; for AML the association (1.63) with grandparents was of borderline significance (lower 95% CI was 0.98).

**TABLE 3:** SIR for childhood acute lymphoblastic leukemia when family members were diagnosed with ALL, CLL, AML, and CML

	Family history with leukemia															
	ALL				CLL				AML				CML			
	O	SIR	95% CI		O	SIR	95% CI		O	SIR	95% CI		O	SIR	95% CI	
Family history, any	32	**1.92**	**1.31**	**2.71**	46	**1.40**	**1.03**	**1.87**	30	1.36	0.92	1.94	6	0.57	0.21	1.26
First‐degree relatives	12	**3.24**	**1.67**	**5.68**	7	1.27	0.50	2.63	6	1.50	0.54	3.28	2	0.93	0.09	3.43
Father	2	3.66	0.35	13.46	5	1.42	0.45	3.34	1	0.56	0.00	3.19	1	0.98	0.00	5.63
Mother	1	2.16	0.00	12.37	1	0.61	0.00	3.52	4	2.93	0.76	7.57	1	1.43	0.00	8.22
Sibling	9	**3.32**	**1.50**	**6.32**	1	2.58	0.00	14.79	1	1.15	0.00	6.60	0			
Grandparents	1	0.60	0.00	3.46	31	**1.48**	**1.00**	**2.10**	19	1.63	0.98	2.55	2	0.41	0.04	1.51
Cousins	16	**1.88**	**1.07**	**3.06**	1	2.01	0.00	11.50	1	0.42	0.00	2.42	1	0.75	0.00	4.29
Uncles/aunts	3	0.98	0.19	2.91	7	1.05	0.42	2.18	6	1.29	0.46	2.83	2	0.81	0.08	2.98

ALL, acute lymphoblastic leukemia; AML, acute myeloid leukemia; CI, confidence intervals; CLL, chronic lymphocytic leukemia; CML, chronic myeloid leukemia; O, observed; SIR, standardized incidence ratio.

Bolding indicates that that 95%CI does not include SIR 1.00.

Familial risks for childhood ALL were assessed when family members were diagnosed with any other types of cancer (Table 4). Risk for childhood leukemia was associated with any cancer in first‐degree relatives for ALL (1.16) and AML (1.32). The individual cancers with significant association with ALL were leukemia (2.13) and endometrial cancer (1.68). Association with colorectal (particularly rectal) and testicular cancers were of borderline significance (lower 95% CI was 0.98). Among female ALL patients, the association with testicular cancer was significant (3.21, 1.65‐5.63; N = 12); for female ALL patients also associations with melanoma (1.67, 1.09‐2.45; N = 26) and skin squamous cell carcinoma (1.94, 1.03‐3.32; N = 13) were significant. Childhood CLL was associated with rectal (and colorectal) cancer (9.57). Childhood AML was associated with pancreatic (2.47) and bladder (2.70) cancers, and of borderline significance (lower 95% CI was 0.96) with prostate cancer.

**TABLE 4:** SIR for childhood leukemia when first‐degree relatives were diagnosed with cancer

	ALL				CLL				AML				CML			
Cancer in probands	O	SIR	95% CI		O	SIR	95% CI		O	SIR	95% CI		O	SIR	95% CI	
Upper aerodigestive tract	17	1.33	0.77	2.13	0				5	1.49	0.47	3.51	0			
Esophagus	5	1.28	0.40	3.01	0				0				1	3.62	0.00	20.75
Stomach	14	1.24	0.68	2.09	1	3.09	0.00	17.73	5	1.30	0.41	3.05	0			
Colorectal	71	1.26	0.98	1.59	5	**4.35**	**1.37**	**10.23**	18	1.13	0.67	1.79	2	0.50	0.05	1.85
Colon	40	1.15	0.82	1.57	1	1.37	0.00	7.83	15	1.50	0.84	2.48	2	0.80	0.08	2.95
Rectum	31	1.44	0.98	2.04	4	**9.57**	**2.49**	**24.74**	3	0.51	0.10	1.50	0			
Liver	11	1.09	0.54	1.95	1	4.30	0.00	24.65	3	0.96	0.18	2.85	0			
Pancreas	14	1.29	0.70	2.17	1	4.11	0.00	23.53	8	**2.47**	**1.06**	**4.89**	0			
Lung	45	1.33	0.97	1.78	1	1.43	0.00	8.17	7	0.71	0.28	1.48	3	1.28	0.24	3.78
Breast	102	1.06	0.86	1.28	0				27	1.20	0.79	1.74	9	1.64	0.74	3.13
Cervix	12	1.07	0.55	1.88	0				2	0.77	0.07	2.83	0			
Endometrium	19	**1.68**	**1.01**	**2.64**	0				2	0.60	0.06	2.21	1	1.23	0.00	7.05
Ovary	10	1.08	0.51	1.99	0				3	1.22	0.23	3.61	0			
Prostate	94	1.09	0.88	1.33	1	0.72	0.00	4.12	31	1.42	0.96	2.02	8	1.49	0.63	2.94
Testis	15	1.75	0.98	2.90	0				0				0			
Kidney	6	0.44	0.16	0.96	0				6	1.58	0.57	3.46	2	2.15	0.20	7.91
Urinary bladder	18	0.91	0.54	1.45	0				15	**2.70**	**1.51**	**4.47**	1	0.73	0.00	4.20
Melanoma	46	1.28	0.93	1.70	0				10	1.40	0.67	2.59	3	1.77	0.33	5.23
Skin	19	1.20	0.72	1.88	0				8	1.88	0.80	3.71	1	0.89	0.00	5.09
Nervous system	28	1.09	0.72	1.57	0				9	1.64	0.74	3.12	2	1.60	0.15	5.87
Thyroid gland	9	1.17	0.53	2.24	0				2	1.28	0.12	4.71	0			
Endocrine glands	9	0.78	0.35	1.48	0				4	1.55	0.40	4.02	0			
Connective tissue	6	1.39	0.50	3.04	0				1	1.06	0.00	6.07	0			
Hodgkin disease	9	1.83	0.83	3.49	0				1	1.02	0.00	5.85	0			
Non‐Hodgkin lymphoma	16	0.93	0.53	1.52	0				7	1.69	0.67	3.50	1	1.02	0.00	5.87
Myeloma	7	1.33	0.53	2.75	0				2	1.39	0.13	5.12	0			
Leukemia	38	**2.13**	**1.51**	**2.93**	0				6	1.43	0.52	3.14	0			
Primary unknown	8	0.78	0.33	1.54	1	4.74	0.00	27.17	4	1.36	0.35	3.51	0			
All	657	**1.16**	**1.08**	**1.26**	11	1.19	0.59	2.13	188	**1.32**	**1.14**	**1.52**	34	0.98	0.68	1.37

AL, acute lymphoblastic leukemia; AML, acute myeloid leukemia; CI, confidence intervals; CL, chronic lymphocytic leukemia; CML, chronic myeloid leukemia; O, observed; SIR, standardized incidence ratio.

Bolding indicates that that 95%CI does not include SIR 1.00.

## 4 DISCUSSION

The results showed that in childhood ALL patient's family history of ALL was rare, 1.4% (including twins). While this may sound a low percentage it is not very different from other rare cancers in Sweden, including Hodgkin lymphoma, or testicular and esophageal cancers for which the rates are around 1.6‐1.7% [26]. A total of three twin pairs were concordant for ALL and the familial risk was excessive, 119, in line with earlier literature [2, 27]. Interestingly, all the present twins were females, as were three other ALL patients whose cotwin was diagnosed with other leukemia. In a previous Swedish–Finnish study, the twin risk was 162, based on four twin pairs, one of which was male‐male [27]. Whether the female preference among concordant twins diagnosed with ALL is a general finding could not be concluded, as the summary of the historical small twin studies, collected by Greaves and coworkers, did not report sex of the affected twins [28]. In a private communication, Professor Greaves kindly checked the gender data on 22 pairs with concordant ALL; of these pairs, 12 were females and 10 males. He also pointed out an old report on seven males and five females ALL pairs [29]. Thus, a gender preference is unlikely.

The familial risk for ALL in singleton siblings was 3.34, in agreement with the previous study [27]. Childhood leukemia of CLL, AML, and CML showed no concordant family history considering even adult forms of these leukemias. Yet for ALL, the novel association with adult probands provide support to the heritable component; in 23 of 32 patients, the family history was for adult ALL probands (SIR 1.64). Moreover, childhood ALL was increased (SIR 1.40) when adult family members were diagnosed with CLL. For ALL, cousin probands contributed to the familial risk, while for CLL grandparents showed the largest contribution. The difference in types of relatives was due to the structure of the family data as siblings could be defined in the offspring generations only, not in the parental generation (see Materials and Methods).

To our knowledge, associations of specific childhood leukemia with any other cancer have been limited to an early version of the Swedish Family‐Cancer Database while a US study was negative [14, 30]. In the Swedish study, an association with testicular cancer was reported. In the present study, we had five more cases of testicular cancer but the SIR had decreased to 1.75 from the earlier 3.12; yet the association remained significant for female ALL patients. The origin of testicular cancer is believed to be in the fetal gonocytes; thus, the gestational origin may provide mechanistic links to ALL [31, 32]. In the present analysis, associations with leukemia and endometrial cancer were significant and with rectal cancer of borderline significance. Among female ALL patients, the associations with melanoma and skin squamous cell carcinoma were significant. While these skin cancers are sensitive to ultraviolet radiation‐induced DNA damage, many of the rare predisposing genes for ALL, such as those related to Fanconi anemia, Ataxia telangiectasia, and Bloom syndrome encode DNA repair enzymes and the defects manifest in skin cancers [3]. Childhood CLL was associated with rectal cancer, and childhood AML was associated with pancreatic and bladder cancers, and of borderline significance with prostate cancer. While endometrial, rectal, pancreatic, and bladder cancers are features of Lynch syndrome with defects in the mismatch repair genes, leukemias are not considered part of the syndrome, except for the related but rare early onset mismatch deficiency syndrome [3, 33].

The basic limitation on this and other studies on childhood leukemia is due the rareness of the disease, whereby statistical power is limited. In the same vein, positive findings in the literature are rare, and thus, support to the present positive findings can hardly be found by referring to published studies. We cannot exclude that some of the positive associations were the result of chance. Thus, the results are descriptive lacking solid mechanistic explanations. The strengths of the study are the nation‐wide access to cancer and family data of high quality, which is one of the unique combinations globally.

In conclusion, we showed that 1.4% of childhood ALL patients had a family history of ALL, in line with familial proportions for other rare cancers. The novel results included association of childhood ALL with adult ALL and a shared risk between cousins. While other types of childhood leukemia were rare and no concordant associations were found even with adult leukemia, childhood ALL was associated with adult CLL. Childhood ALL was associated with endometrial cancer, and female ALL patients showed increased risk when family members were diagnosed with testicular and skin cancers and melanoma. Childhood CLL was associated with rectal cancer, and childhood AML was associated with pancreatic and bladder cancers.

The study was supported by the European Union's Horizon 2020 research and innovation program, Grant No. 856620, the Swedish Research Council (2016‐01176; 2018–02400), and the ALF project grant, Region Skane/Lund University, Sweden.

## AUTHOR CONTRIBUTIONS

Kristina Sundquist, Jan Sundquist provided the data; Xinjun Li carried out analyses; Kari Hemminki and Asta Försti planned the study and wrote the manuscript. All authors have approved the final manuscript.

## CONFLICT OF INTEREST

The authors declare that they have no conflict of interest.

## ETHICS

The guidelines of the Helsinki Declaration were followed. The study was approved by the Regional Ethical Review Board in Lund, Sweden (2012/795).

## Supporting information

SUPPORTING INFORMATION

## Data Availability

The data were obtained with a special agreement from the Swedish National Board of Health and Welfare. Any applications for these data should be addressed to this authority.
