# Supplementary material for: Family history of any cancer for childhood leukemia patients in Sweden
Source: EJHaem. 2021 May 3;2(3):421–7. doi: 10.1002/jha2.166 (PMC9175698; doi:10.1002/jha2.166)
Supplement: Supplementary file 1 — SUPPORTING INFORMATION [file JHA2-2-421-s001.docx]

| **Supplementary Table 1. Characteristics of study population and number of cases of leukemia in childhood in Sweden, 1958-2015** | | |
| --- | --- | --- |
|  | No. | % |
| Population | 8514305 |  |
| Gender |  |  |
| Males | 4371909 | 51.3 |
| Females | 4142396 | 48.7 |
| Birth year |  |  |
| 1938-49 | 1147300 | 13.5 |
| 1950-59 | 1055133 | 12.4 |
| 1960-69 | 1182109 | 13.9 |
| 1970-79 | 1095007 | 12.9 |
| 1980-89 | 1084358 | 12.7 |
| 1990-15 | 2950398 | 34.7 |
|  |  |  |
| Cases of Childhood leukemia (aged < 20 years) | 4461 |  |
| Leukemia subtype |  |  |
| Acute lymphoblastic leukemia | 2859 | 64.1 |
| Chronic lymphoblastic leukemia | 24 | 0.5 |
| Acute myeloid leukemia | 556 | 12.5 |
| Chronic myeloid leukemia | 106 | 2.4 |
| Gender |  |  |
| Males | 2465 | 55.3 |
| Females | 1996 | 44.7 |
| Birth year |  |  |
| 1938-49 | 117 | 2.6 |
| 1950-59 | 312 | 7.0 |
| 1960-69 | 726 | 16.3 |
| 1970-79 | 794 | 17.8 |
| 1980-89 | 801 | 18.0 |
| 1990-15 | 1711 | 38.4 |
| Age at diagnosis (years) |  |  |
| 0-4 | 1978 | 44.3 |
| 5-9 | 1145 | 25.7 |
| 10-14 | 714 | 16.0 |
| 15-19 | 624 | 14.0 |
